# Isolation, identification of entomopathogenic nematodes with insights into their distribution in the Syrian coast regions and virulence against *Tuta absoluta*

**DOI:** 10.2478/jofnem-2023-0056

**Published:** 2023-11-30

**Authors:** Mai Ali, Nada Allouf, Mohammad Ahmad

**Affiliations:** Department of Plant Protection, Faculty of Agriculture, Tishreen University, Latakia, Syria

**Keywords:** *Heterorhabditis*, *Steinernema*, Identification, Distribution, Biological control, Syria

## Abstract

The occurrence and distribution of entomopathogenic nematodes (EPNs) in the Syrian coast regions remain relatively uncharted. To address this gap in our knowledge, an extensive survey of these ecosystems was essential. This study aims to isolate and identify EPNs from diverse ecosystems within the coastal regions. The distribution of EPNs in cultivated and natural environments was analyzed according to habitat, altitude, and sampling season factors. Between 2017 and 2020, EPNs were recovered from 27 out of 821 soil samples (3.28%) and collected from 24 out of 375 sampling sites (6.4%). Based on morphological, morphometric, and molecular (ITS) characteristics, four EPN species were identified: *Heterorhabditis indica* (51.85%), representing the first report of its occurrence in the coastal regions, *H. bacteriophora* (33.33%), *H. pakistanense* (7.4%), which is also reported for the first time in Syria, and *Steinernema affine* (7.4%). There were statistical differences in the abundance and recovery frequency of EPNs in each type of habitat. Additionally, there were statistical differences in the altitude and sampling season recovery frequency.

Co-inertia analysis revealed correlation between the distribution and occurrence of EPNs in vegetation habitats, altitude, and sampling seasons, as well as some soil characteristics. *H. indica* and *H. bacteriophora* were associated with citrus orchards, low-altitude ranges, moderate organic matter, and acidic soil. More specifically, *H. indica* isolates were correlated with olive orchards, vegetable fields, autumn season, and clay, sandy, and sandy loam soils. Meanwhile, *H. bacteriophora* isolates were correlated with tobacco fields, grasslands, alkaline pH, spring season, silty loam, and clay loam soils. *H. pakistanense* was linked to pear orchards, vineyards, moderate pH, and low organic matter. *S. affine* occurred in walnut orchards, silty soil, higher altitudes, and winter season.

The virulence levels of three native EPN isolates (*S. affine*, *H. indica* and *H. bacteriophora*) were evaluated against 3^rd^ and 4^th^ instar larvae (outside and inside mines) and pupae of *T. absoluta*, a destructive pest in Syria. All three native EPN species exhibited ability to infect and kill the insect, with observed significant differences in their virulence. This study provides an understanding of EPN occurrence, distribution, and their potential for application in sustainable pest control strategies in Syria.

Intensive farming worldwide led to an overuse of chemical pesticides, exacerbating their adverse effects on humans and the environment. As a result, researchers have shifted their focus to biological approaches to pest management to reduce dependence on chemical pesticides. They have worked to discover more natural enemies and encourage their usage in Integrated Pest Management (IPM) programs (Deguine *et al*., 2021). In 2013, the global bio-pesticide market accounted for approximately 5% of the total crop protection market, which is valued at $3 billion (Olson, 2015). This segment of the industry is growing at a compounded annual growth rate (CAGR) of 8.64%, (Olson, 2015). It is expected that by 2025, the sales of bio-pesticides will exceed $8.5 billion (Khakimov *et al*., 2020).

In recent years, entomopathogenic nematodes (EPNs) have gained increasing attention as an effective non-chemical alternative for controlling insect pests, specifically those belonging to the families Heterorhabditidae (Poinar, 1976) and Steinernematidae (Travassos, 1927) (Shapiro-Ilan *et al*., 2012). Nematode species in these families are lethal endoparasites that form a mutualistic relationship with symbiotic bacteria from the *Xenorhabdus* and *Photorhabdus* genera, respectively. This mutualistic association is obligate in nature, with each partner depending on the other to complete its life cycle (Sivaramakrishnan and Razia, 2021).

The bacteria are transported by the free-living infective juvenile (IJ) stage of the EPN, as they are located in the anterior part of the *Heterorhabditis* intestine, or, in most of the *Steinernema* spp., in a special intestinal vesicle (Campos-Herrera *et al*., 2013). The IJ act as active vectors, surviving in the soil until they find a target insect host, then entering the host through its natural apertures (mouth, anus, or spiracles) or, in some cases, through the cuticle (Koppenhöfer *et al*., 2020). Symbiotic bacteria are released through defecation or regurgitation into the hemocoel of insects, where the bacterial cells multiply and produce several toxins or secondary metabolites, killing the insect hosts within 24–48 hours by suppressing the immune system and causing toxemia and septicemia (Mollah and Kim, 2020).

It's worth noting that the host defenses and immune reactions in response to EPN infection have been studied only in a few EPN-insect species combinations. Those studies have demonstrated that in *Heterorhabditis*-*Photorhabdus* combinations, the bacteria seem to play a major role in killing the host (Koppenhöfer *et al*., 2020). In *Steinernema-Xenorhabdus* combinations, by contrast, the nematodes play a more active role in contributing to the virulence of the nematode-bacterium complex, releasing venom proteins that include both tissue-damaging and immune-modulating proteins (Lu *et al*., 2017; Chang *et al*., 2019).

In addition to their ability to rapidly kill hosts, EPNs have other advantages that make them the most suitable candidates for development and use as efficient biological control agents (Manochaya *et al.,* 2022), such as movement ability, high virulence, high reproductive potential, wide host range, mass *in-vivo* and *in-vitro* production capability, and safety to vertebrates, plants, and many other non-target organisms (Lacey and Georgis, 2012).

However, it is also essential to consider the factors that influence the occurrence, effectiveness, and persistence of EPNs within ecological systems. These factors include abiotic elements, such as climate, soil pH, texture, and structure (Stuart *et al*., 2015), as well as biotic factors, particularly the soil environment, which houses diverse communities of interconnected flora and fauna, creating complex trophic relationships (Campos-Herrera *et al*., 2012). Within this intricate ecological framework, the interspecific competition among EPN species assumes significant importance due to the potential impacts of biological control applications involving exotic or native nematodes on native nematode communities (Stuart *et al*., 2015). In this context, Duncan *et al*. (2003) observed the suppression and partial displacement of native EPNs (*Steinernema diaprepesi* Nguyen & Duncan, 2002, *H. bacteriophora* Poinar, 1976, *H. indica* Poinar, Karunakar & David, 1992, and *H. zealandica* Poinar, 1990) following the application of exotic *S. riobrave* Cabanillas, Poinar and Raulston to control the root weevil *Diaprepes abbreviatus* in Florida citrus.

Thus, the detection and identification of native EPN species has become a priority in knowing and understanding their distribution within the targeted ecological systems, as well as enhancing their persistence (Sun *et al.*, 2021). Native EPN isolates, however, are expected to be well adapted to local conditions, ideally including the pest itself (Labaude and Griffin, 2018). Globally, several surveys were conducted to isolate EPN species and identify their geographic distribution (Bhat *et al.*, 2020). These investigations found 102 *Steinernema* species, 22 *Heterorhabditis* species, and one *Neosteinenema* species (Hazir *et al*., 2022).

In Syria, the first comprehensive survey of the entire country was conducted by Canhilal *et al*. (2006). All five of their isolates tested positive for *H. bacteriophora*. Notably, their findings indicated the absence of EPN in the soils of the Syrian coastal regions (Latakia and Tartus governorates). Subsequently, two studies of some agricultural systems within the governorate of Latakia were carried out, identifying two species of EPN: *H. bacteriophora* and *S. cubanum* Mráček, Hernández and Boemare, 1994 (Mosalam, 2009; Zeini *et al*., 2019).

The majority of coastal regions, spanning 4,200 km^2^, remain largely uncharted in our understanding of EPN's geographical occurrence, distribution, and associated environmental factors. Furthermore, there is a need to determine whether potential isolates can effectively control local pests. To address these knowledge gaps and provide initial insights, we conducted an extended survey covering both cultivated and natural environments in the coastal regions by collecting 821 soil samples from 375 sites between the years 2017 and 2020.

The objectives of the present study were (1) to investigate the natural occurrence of EPN in the soils of the Syrian coast regions and identify their species through an integrative taxonomic approach based on morphological, morphometric, and molecular (ITS) data; (2) to understand their occurrence and geographic distribution in correlation with several factors, such as habitat, soil type, pH, organic matter (%), altitude, and sampling season; and (3) to evaluate the virulence of the detected native EPN isolates against *Tuta absoluta* (Meyrick, 1917) (Lepidoptera: Gelechiidae).

## Material and Methods

### Survey scheme, sampling methods and soil properties analyses

**Figure 1:**
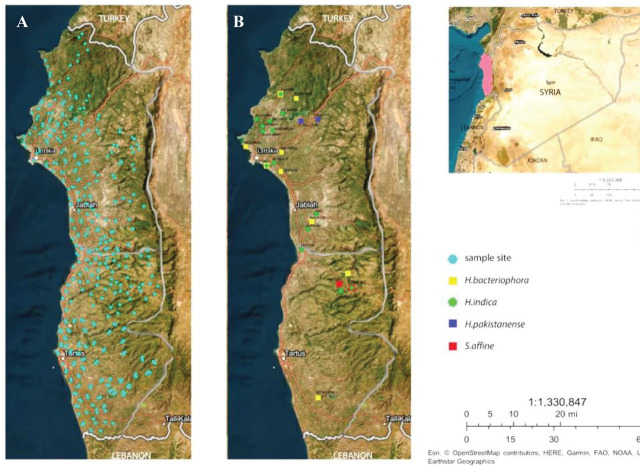
Distribution map of soil sample sites and entomopathogenic nematodes in Syrian coastal regions. A: Soil sample sites. B: Sites positive for entomopathogenic nematodes. Those marked with green denote *H. indica*, yellow denotes *H. bacteriophora*, blue denotes *H. pakistanense*, and red denotes *S. affine*.

A total of 821 soil samples were collected randomly from 375 sites in Syrian coastal regions (Fig. 1). The survey was primarily conducted during the spring and autumn months, with 375 and 319 samples collected during those months, respectively, and also during the summer and winter seasons, with 71 and 56 samples, respectively, collected during those months between the years 2017 and 2020.

In order to survey various ecological habitats, soil samples were collected from both natural and cultivated ecosystems at altitudes ranging from 0 to 1,200 m above sea level and from different habitats identified based on ecological similarities, namely coniferous forests (*Pinus* spp., *Cedrus* spp., *Chamaecyparis* spp., *Juniperus* spp., and several kinds of trees); (*n* = 57); broadleaf forests (*Quercus* spp., *Acer* spp., *Arbutus* spp., *Ceratonia* spp., *Pistacia* spp., and several kinds of trees) (*n* = 62); orchards (*Citrus* spp. (*n*=33); *Olea* spp. (*n* = 37); *Juglans* spp. (*n* = 25); *Vitis* spp. (*n*=25); and *Pyrus* spp. (*n*=23). They were also collected from several kinds of fruit orchards (e.g., *Malus pumila*, *Prunus avium*) (*n*=55); vegetable fields (*Solanum melongen, Solanum tuberosum*, *Cucurbita pepo*, *Abelmoschus esculentus*, and several other kinds of vegetables) (*n* = 40); oil crops (*Zea mays*, *Arachis* spp., *Helianthus* spp.) (*n*=39); leguminous crops (*Cicer* spp., *Pisum* spp., *Phaseolus* spp.( (*n* = 48); leafy vegetable fields (*Spinacia oleracea*., *Brassica oleracea*., *Petroselinum crispum* spp., *Allium cepa*., *Brassica oleracea* var. *acephala*, and several other kinds of plants) (*n* = 43); tobacco fields (American tobacco, aromatic tobacco, and semi-aromatic tobacco) (*n* = 24); greenhouses (*Solanum lycopersicum*, *Capsicum annuum*, *Cucumis sativus*, and ornamental plant (*n* = 86); fodder crops (*Medicago sativa*, *Sorghum bicolor*, *Hordeum* spp, *Vicia* spp) (*n* = 48); grassland (*n*=45); riverbanks (*n*=31); medicinal and aromatic plants (*Melissa* spp., *Salvia officinalis*, *Salvia rosmarinus*, *Thymus* spp.) (*n* = 39); and environments including sea coast (*Palmae* spp., *Hibiscus* spp., sand dunes) (*n* = 34) and public parks (*Jacaranda* spp., *Azadirachta* spp., *Dodonaea* spp., *Cercis* spp.) (*n* = 27).

At each site, the soil sample (approximately 2000 g) was composed of five random sub-samples taken with a hand shovel to a depth of 30 cm over an area of 10 m^2^. The composite samples were placed in polyethylene bags to prevent water loss, and these were kept in coolers as recommended by Kaya and Stock (1997). The soil samples were transported to the Plant Protection Research Laboratory in the Faculty of Agriculture at Tishreen University for the isolation of EPNs. Soil physico-chemical analyses, including soil type, organic matter content (%), and pH, were carried out for the EPN-positive samples only in the laboratories of the Scientific Agricultural Research Center in Latakia. These characteristics, as well as other data from the positive sampling sites, are indicated in Table 1.

**Table 1:** Locality, coordinates, altitude, sampling date, habitat, and soil characteristics of positive samples.

**Species**	**Site/Location**	**GenBank accession no.**	**Coordinates**	**Altitude**	**Sampling date**	**Habitats**	**Soil types**	**pH**	**Organic matter %**
*H. indica*	Zeghreen / Latakia	OP221993	35°43′39″N 35°52′39″E	40 m	Autumn	Olive field	Sandy	7.3	3.39
*H. indica*	Al-Hinadi / Latakia	OP221988	35°51′48″N 35°30′12″E	36 m	Autumn	Citrus orchard	Silty loam	7.5	1.4
*H. indica*	Ein-Albida / Latakia	OP221987	35°39′26″N 35°35′18″E	207 m	Autumn	Citrus orchard	Silty loam	7.5	5.19
*H. indica*	Al-Meherfha/Latakia	OP221992	35°36′12″N 35°51′20″E	214 m	Autumn	Olive orchard	Sandy loam	6.1	2.3
*H. indica*	Al-Shamiea /Latakia	OP221989	35°38′22″N 35°48′30″E	61 m	Autumn	Citrus orchard	Silty loam	6.5	3.72
*H. indica*	Fattiro /Latakia	OP221991	35°37′19″N 35°15′24″E	138 m	Autumn	Olive orchard	Clay	6.2	2
*H. indica*	Kersana/Latakia	OP221983	35°37′18″N 35°49′40″E	53 m	Autumn	Olive orchard	Sandy loam	6.0	5.72
*H. indica*	Al-Basa/Latakia	OP221984	35°30′14″N 35°52′15″E	46 m	Autumn	Vegetables	Silty loam	6.5	2.33
*H. indica*	Al-Borjan /Latakia	OP221986	35°17′34″N 35°58′42″E	47 m	Autumn	Vegetables	Sandy loam	6.1	3.66
*H. indica*	Al-Meghreet /Latakia	OP221990	35°63′17″N 35°49′53″E	81 m	Spring	Citrus orchard	Silty loam	6.4	2.9
*H. indica*	Al-Hwiz/Latakia	OP221996	35°20′27″N 36°00′02″E	108 m	Spring	Grassland	Sandy loam	5.2	2.26
*H. indica*	Hraeson / Tartus	OP221985	35°15′01″N 35°57′01″E	15 m	Spring	Citrus orchard	Silty loam	8.1	2.73
*H. indica*	Fneiteq /Tartus	OP221994	35°06′51″N 36°07′17″E	597 m	Autumn	Walnut orchard	Silty loam	8.1	1.73
*H. indica*	Karm-Alten/Tartus	OP221995	35°07′01″N 36°06′20″E	580 m	Spring	Grassland	Sandy	7.5	3.02
*H. bacteriophora*	Al-Snobr /Latakia	OP222916	35°28′45″N 35°53′09″E	26 m	Spring	Grassland	Clay loam	5.8	6.2
*H. bacteriophora*	Zeghreen /Latakia	OP222020	35°43′45″N 35°52′57″E	46 m	Autumn	Citrus orchard	Clay loam	6.7	4.72
*H. bacteriophora*	Al-Shelfatea/Latakia	OP222019	35°32′54″N 35°53′53″E	41 m	Autumn	Citrus orchard	Silty loam	7.1	3.3
*H. bacteriophora*	Al-Serskiea/Latakia	OP222018	35°42′33″N 35°55′36″E	85 m	Autumn	Vegetables	Sandy loam	6.7	5.39
*H. bacteriophora*	Ras-Alein /Latakia	OP222015	35°17′38″N 35°57′53″E	32 m	Spring	Tobacco field	Silty loam	6.0	1.46
*H. bacteriophora*	Al-Basa /Latakia	OP222017	35°29′34″N 35°50′43″E	4 m	Autumn	Citrus orchard	Silty loam	7.7	2.67
*H. bacteriophora*	Dimsarkho /Latakia	OP222012	35°33′07″N 35°46′26″E	15 m	Autumn	Tobacco field	Silty loam	5.9	3.06
*H. bacteriophora*	AL-Kawkae/Tartus	OP222014	35°09′24″N 36°06′47″E	486 m	Spring	Grassland	Clay loam	6.3	2.06
*H. bacteriophora*	Bet-shofan /Tartus	OP222013	34°45′35″N 36°00′44″E	70 m	Spring	Citrus orchard	Silty loam	7.8	1.25
*H. pakistanense*	Al-Zobar /Latakia	OP235514	35°37′57″N 36°00′12″E	213 m	Autumn	Pear orchard	Sandy loam	6.8	1.86
*H. pakistanense*	Al-Bahloliea/Latakia	OP235513	35°38′09″N 35°57′40″E	179 m	Summer	Vineyard	Sandy loam	7.1	1.3
*S. affine*	Amodeyha/Tartus	OM350119	35°06′10″N 36°07′57″E	759 m	Spring	Walnut orchard	Silty loam	6.1	6
*S. affine*	Fneitiq/Tartus	OL437016	35°06′02″N 36°08′51″E	604 m	Winter	Grass land	Silty	6.4	4.1

### Isolation of EPNs with *Galleria mellonella*

EPNs were isolated from soil samples using the “Galleria-bait method” (Bedding and Akhurst, 1975). Two 500-g sub-samples from each composite sample were placed in separate clean plastic containers with ten larvae of *Galleria mellonella* (Lepidoptera: Pyralidae). The containers were then covered with lids, turned upside down, and kept in the dark at room temperature for 14 days. This duration was chosen due to the presence of other entomopathogens within the tested soil samples. During this period, the isolation process was repeated two to three times. In each round, new *G. mellonella* larvae were added to the containers, and the soil moisture was adjusted as needed. The larvae in the containers were checked every two days (Kaya and Stock, 1997). Dead larvae that exhibited marks of infection with EPN were collected, washed twice in distilled water, and placed in a modified White trap (White, 1927). The emerging IJ were cleaned and used to confirm Koch's postulate, then stored in tissue culture flasks at 12 ºC.

### Morphological and morphometric characterization

Ten last-instar larvae of *G. mellonella* (L.) were exposed to ca. 300 IJ in a 9-cm-diameter petri dish (lined with two moist filter papers (Whatman No. 1, Maidstone, UK) (Nguyen, 2007). The cadavers were dissected in Ringer's solution (9.0 g Sodium chloride, 0.4 g Potassium chloride, 0.4 g Calcium chloride, 0.2 g Sodium carbonate, and 1000 ml Sterile distilled water) after four to five days post-infection to obtain both males and amphimictic females. In this research, we specifically studied the characteristics of the males, as they play a significant role in species identification, in addition to third-stage IJ that were collected within the initial two days following their emergence from the cadavers. For the light microscope observations and assessment, 20 living specimens were examined from each life stage of males and IJ. Additionally, specimens were killed and fixed in TAF (7ml Formalin, 2ml Triethanolamine, 91 ml water) (Kaya and Stock, 1997). Morphological traits and morphometric measurements were observed under a light microscope equipped with software (Optika Proview, Optika, Ponteranica, Italy) using 4×, 10×, 40×, and 60× objectives.

The following characteristic were studied and measured, as mentioned by Nguyen and Hunt (2007): total body length (L), maximum body diam (MBD), distance from anterior end to excretory pore (EP), distance from anterior end to nerve ring (NR), distance from anterior end to base of the esophagus (ES), tail length (T), anal body diam (ABD), with the ratios a = L/MBD, b = L/ES, c = L/T, D = EP/ES, and E = EP/T. Additionally for males, length of the spicules (SL), length of the gubernaculum (GL), and Testis reflexion (TR), SW% = SL/ABD×100, and GS% = GL/SL×100, were measured. To examine the bursa of males, living males were transferred at random into a small drop of lactophenol on a hot plate at 65 °C. After 30 min, one of the males was transferred to a big drop of lactophenol on a glass slide. The anterior part of the body was removed, and then the cover glass was placed on the posterior part of the male, which was moved slowly by means of a needle in order to rotate the nematode posterior region so as to present a ventral view. Thirty bursae were observed, and the distribution of bursal rays was studied (Nguyen, 2007).

### DNA extraction and molecular identification of nematodes

Total genomic DNA was extracted from a single hermaphroditic using a DNeasy Blood and Tissues Kit (QIAGEN, Venlo, Netherlands) following the manufacturer's protocol. The entire internal transcribed spacer region (ITS) of rDNA was amplified using the primer TW81 (5′-GTTTCCGTAGGTGAACCTGC-’3) for ward and AB28 (5′-ATATGCTTAAGTTCAGCGGGT-’3) reverse (Torrini *et al*., 2020). The thermocycle protocol for the PCR was one cycle at 94 ºC for 2 min and 35 cycles at 94 ºC for 1 min, followed by 55 ºC for 45 s and 72 ºC for 2 min, then 72 ºC for 10 min. 10μL of the PCR product was loaded on a 1.2% agarose gel stained with 0.5 μg/ml ethidium bromide, with 1X TBE buffer running in isolation under voltage 80 v for two hours. A 100-bp DNA ladder was used for approximate quantification of the DNA product. The gel was visualized under ultraviolet light and photographed using a BioDocAnalyze gel documentation system (Analytik Jena, Jena, Germany). The target bands were purified using gel purification kit NucleoSpin Extract II Kit (Thermo Fisher Scientific, Waltham, MA, USA). The purified PCR products were sequenced by Macrogen Inc. (Amsterdam, Netherlands). The sequences were further analyzed using BioEdit and Finch TV to trim the low-quality bases, then submitted to GenBank to get the accession number. Sequence verification was performed a BLAST (Basic Local Alignment Search Tool) to verify the identity of our sequences by comparing them with the reported ones from NCBI database. For phylogenetic analyses, corresponding ITS sequences of respective EPN were retrieved from the NCBI database and aligned using ClustalW. The evolutionary tree was deduced with maximum likelihood (ML) methods. The phylogenetic tree was constructed using MEGA-X, and cladograms were boot-strapped 1,000 times for statistical validation. The branches representing bootstrap support values lower than 50% were not shown. *Caenorhabditis elegans* (FJ58900.1) was used as an out-group (Kumar *et al*., 2018; Tamura *et al*., 2004).

### Virulence of EPNs against tomato leaf miner *T. absoluta*

Colonies of *T. absoluta* were originally collected from infested tomato greenhouses in Tartus governorate. The insects were reared on potted tomato plants (*Solanum lycopersicum* L, cv. Mandloun, 12 cm in diameter, 6–8 weeks old) within a large wooden rearing cage (210 × 120 × 240 cm) maintained at 25 °C, with a 16:8 h (L:D) photoperiod and 45–88% relative humidity (RH) (Ben Husin and Port, 2021). In this research, the choice of target instar larvae and pupae of *T. absoluta* for the assays was made based on their developmental stage, which was determined using developmental time data and morphological descriptions from a previous study (currently unpublished).

The virulence of native isolates of EPNs was evaluated against the third and fourth instar larvae (both outside leaves and inside mines), as well as pupae of *T. absoluta*. For that, three native isolates of EPNs (*S. affine* M.313, *H. indica* F., and *H. bacteriophora* H.) were used. The virulence experiments were carried out using 24-well tissue culture plates (3.14 cm^2^ surface area/well) lined with circular filter paper and tomato leaf discs, as described by Türköz and Kaşkavalci (2016). A total of six larvae per culture plate were considered as one replicate. Each treatment had four replicates, each containing 24 larvae. The plates were then sealed with Parafilm and placed into an incubator at 18 °C in the dark. The insect mortality was observed and recorded 48 hours after infection in all the conducted assays. Four replicates (*n* = 24) for each treatment, treated with sterile water, served as control.

For the virulence assay in outside leaves, each larva from the target instar was transferred individually into one well. Then nematodes of each species were applied at dose levels of 1, 5, 10, 15, 20, 25, and 50 IJ per target tomato leaf miner larva.

For the virulence assay inside the mines, the LD_50_ inoculation rates for the most efficient species from the previous assay (*S. affine*. M 313 and *H. indica.* F) were used. The third-instar larvae were transferred to a culture plate and given ca. 10 minutes to establish their mines. Then, the LD_50_ of third-instar larvae from the outside-leaves assay for *S.affine* and *H. indica* were separately applied to each plate. A similar experimental design was followed for the assay of the fourth-instar larvae inside mines, with the applied inoculation rate of nematodes corresponding to the LD_50_ for the fourth-instar larvae from outside leaves.

In the pupal experiment, LD_50_ of third-instar larvae from outside leaves of *S. affine* and *H. indica* were used. Each pupa was individually placed into a well containing 10% moistened sterilized sand, the inoculation rates were then separately added to each well for each species in the plate (replicates = 4, *n* = 24).

### Data analysis

To explore the ecology of native EPN isolates, the occurrence was assessed as recovery frequency (no. positive samples/no. total samples) and abundance (no. positive sites/no. total sites) (Campos-Herrera *et al*., 2007), expressed as a percentage. The distribution of the positive samples in each site was adjusted to a negative binomial distribution (Ludwig and Reynolds, 1988). The occurrence of EPN was related to vegetation habitat, altitude, and sampling seasons. To study the influence of those variables, Crosstabs and Pearson's v2 tests at *P*-value = 0.05 were carried out to assess the significant differences in EPN occurrence.

To assess the impact of vegetation, habitat and some abiotic variables on the occurrence and distribution of EPN within the Syrian coastal ecosystems, various types of variables were defined (Table 2), including (i) habitat; (ii) soil physico-chemical characteristics such as type, pH, and organic matter content (%); (iii) altitude; and (iv) sampling season. A co-inertia analysis (COIA) was then applied between EPN data in correspondence to habitat and abiotic variable data (Dray *et al*., 2003). The multivariate analyses and graphical representations were conducted using the ade4 library (Dray and Dufour, 2007).

**Table 2:** Vegetation habitat and abiotic factors with their corresponding codes.

**Habitat**	**Code-Hab**	**Soil characteristics**	**Code**	**Altitude and Season**	**Code**
Citrus orchard	Hab_1_	Soil type	C Sa Si SaL SiL CL	Altitude	Alt_1_ Alt_2_ Alt_3_ Alt_4_
Olive orchard	Hab_2_	Clay Sand Silty Sandy loam Silty loam Clay loam		0–200 m 200–400 m 400–600 m 600–800 m	
Grassland	Hab_3_				
Vegetables	Hab_4_			Season	
Walnut orchard	Hab_5_	pH <6.5 = 6.5–7.5 >7.5	pH_1_ pH_2_ pH_3_	Autumn Spring Summer Winter	S_1_ S_2_ S_3_ S_4_
Tobacco field	Hab_6_				
Pear orchard	Hab_7_				
		Organic matter content 1–2% 2–4% 4–6%	OM_1_ OM_2_ OM_3_		
Vineyard	Hab_8_				

Hierarchical cluster analysis (HCA) was performed using various morphometric measurements of males to illustrate the hierarchical relationships among the *Heterorhabditis* species and group them into homogeneous clusters based on the distance matrix. The variables used for the HCA included: the mean values of body length, EP, NR, ES, ABW, T, SL, and GL, as well as the values of (a), (b), (c), and the ratios of D%, E%, SW%, and GS%.

To assess the virulence of some native EPN isolates against the third- and fourth-instar larvae and the pupae of *T. absoluta,* insect mortality was calculated and expressed as a percentage. Mean values of mortality (%) were analyzed by one way ANOVA. Tukey's test was then used for pairwise multiple comparisons between the EPNs isolates according to ANOVA results. Means mortalities of 3^rd^ and 4^th^ instar were compared outside leaves at different IJ concentrations of EPN, and inside mines (for the 3^rd^, 4^th^ instar and the pupae). Differences obtained at *P* < 0.01 were considered significant. Probit analyses of significantly different treatments were performed using Tukey's honestly significant difference (HSD) test. Means ± SD are presented.

All analyses were done using Genstat software and R version 3.3.2 (R Core Team, 2016).

## Results

### Entomopathogenic nematode distribution in relation to abiotic factors and habitat

Entomopathogenic nematodes were isolated from 27 out of 821 soil samples (3.28%) collected from 24 of the 375 sampling sites (6.4%). Among the 27 isolates, 25 were heterorhabditids (3.04%), and two were steinernematids (0.24%) found in the Syrian coastal regions (Fig. 1). The analysis of the frequency of positive samples showed that most of the sites were negative for EPN occurrence. Three out of 24 positive sites had two EPN species, and in all three sites, the various species were recovered from multiple habitats. For instance, in the Fneitiq site, *H. indica* and *S. affine* were recovered from walnut orchard and grassland, respectively. In the Zeghreen site, *H. bacteriophora* and *H. indica* were found in citrus orchards and olive fields, respectively. Finally, in the Al-Basa site, *H.bacteriophora* and *H. indica* were isolated from citrus orchard and vegetable field, respectively.

Table 3 shows the recovery frequency and the abundance of EPN in each habitat with altitude, and sampling season variables. We observed significant differences between vegetation habitats for each species’ recovery frequency and abundance (*x*^2^ = 28.708. df = 9, *P* = 0.01491) and (*x*^2^ = 30.08, df = 10, *P* = 0.01892), respectively. The highest recovery frequency was recorded within citrus orchards (27.27%), followed by grasslands (11.11%) and olive orchards (10.81%). EPN were not found in coniferous or broadleaf forests, leafy vegetables, medicinal or aromatic plants, in fodder Leguminous and oil crop, or in greenhouses, sea coast, riverbanks and public parks. Although EPN abundance did not show significant differences among altitude ranges, the recovery frequency was significantly different (*x*^2^ = 24.154, df = 7, *P* = 0.0138). The highest EPN recovery frequency was detected at 0–200 m above sea level (8.37%). The lowest occurrence of EPN was recorded at 200–400, 400–600, 600–800 m, at 1.79, 1.93, and 1.39 %, respectively. EPNs were not detected at altitudes at > 800 m. EPN abundance was not significantly different among sampling seasons; although their recovery frequency varied significantly (*x*^2^ = 26.73, df = 7, *P* = 0.0474), the highest EPN recovery frequency was recorded in autumn (5.01%), followed by spring (2.4%) and then winter (1.78%). The lowest EPN recovery frequency was recorded in summer (1.4%).

**Table 3:** Distribution of EPNs in Syrian coast region according to the vegetation habitat, altitude and sampling seasons variables.

**Habitat**	**Frequency**	**Abundance**	**No. of identified samples to species**
Coniferous forest	0 ^c^	0 ^b^	0
Broadleaf forest	0 ^c^	0 ^b^	0
Citrus orchards	27.27 ^a^	2.4 ^a^	9
Olive orchards	10.81^b^	1.06 ^a^	4
Walnut orchards	8 ^b^	0.53 ^a^	2
Pear orchards	4.34 ^b,c^	0.26 ^a^	1
vineyard	4 ^bc^	0.26 ^a^	1
Fruit orchards (e.g., cherry, apple)	0 ^c^	0 ^b^	0
Vegetable fields	7.5 ^b^	0.8 ^a^	3
Oil crop	0 ^c^	0 ^b^	0
Leguminous crop	0 ^c^	0 ^b^	0
Leafy vegetables	0 ^c^	0 ^b^	0
Tobacco fields	8.33^b^	0.53 ^a^	2
Riverbanks	0 ^c^	0 ^b^	0
Greenhouses	0 ^c^	0 ^b^	0
Fodder crop	0 ^c^	0 ^b^	0
Grassland	11.11^b^	1.33 ^a^	5
Medicinal and aromatic plants	0 ^c^	0 ^b^	0
Sea coast	0 ^c^	0 ^b^	0
Public parks	0 ^c^	0 ^b^	0
	*P* = 0.01491	*P* = 0.01892	
**Altitude**			
0–200	8.37^a^	19.31	18
200–400	1.79^b^	3.79	3
400–600	1.93^b^	3.94	3
600–800	1.39^b^	2.7	2
>800	0^c^	0	0
	*P* = 0.0138	*P* = 0.1565	
**Season**			
Autumn	5.01 ^a^	10.37	16
Spring	2.4^a,b^	6.33	9
Summer	1.4^b^	1.96	1
Winter	1.78 ^b^	2.12	1
	*P* = 0.0474	*P* = 0.08053	

### Identification of entomopathogenic nematode species

**Figure 2:**
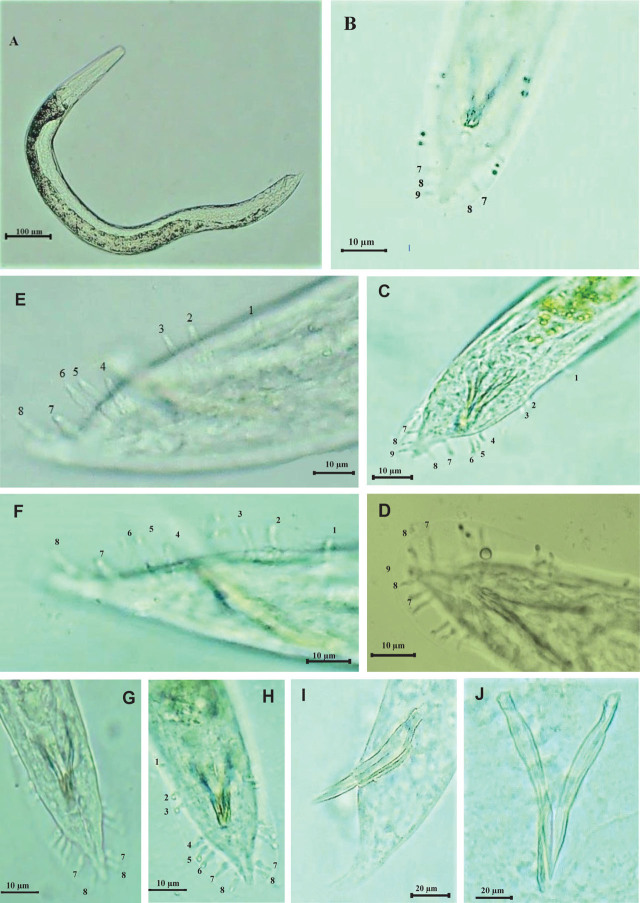
*Heterorhabditis indica* male. A: Male entire body. B–H: Male tail with bursal papillae. B, C: Ventral view of bursa showing papillae (3–2) in the terminal group with some variation. E, F, G, H: Lateral and ventral view of bursa showing papillae (2–2) in the terminal group with some modifications (namely, being short and swollen). I: Lateral view of spicule and gubernaculum. J: Ventral view of spicule and gubernaculum. A = 705 μm. I, J: SL = 40 μm, GL = 19 μm.

**Figure 3:**
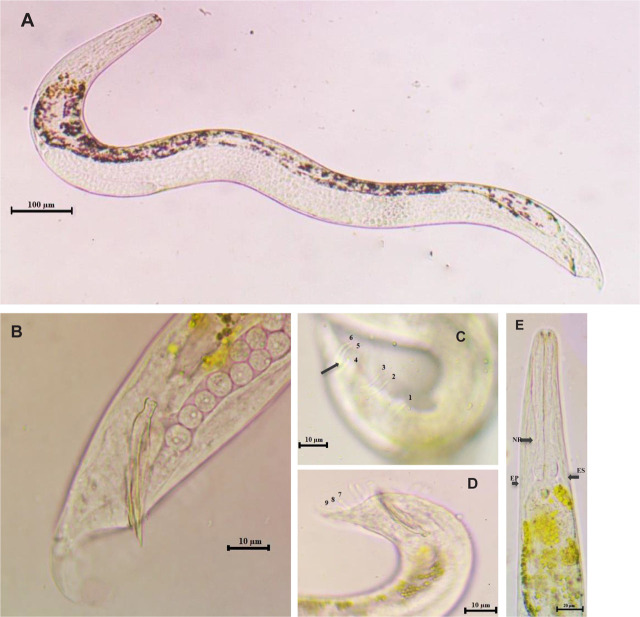
*Heterorhabditis bacteriophora* male. A: Male entire body. B: Male tail with bursal lateral view of spicules and gubernaculum. C: Lateral view of bursa showing the arrangement of papillae, arrow refers to curved outward fourth pair. D: Lateral view of bursa showing the terminal group of bursal papillae pairs (7, 8, and 9). E: Anterior portion; the left arrows refer to the ring position and excretory pore, while the right arrow refers to the end of the esophagus. A = 861 μm. B: SL = 42 μm, GL = 20 μm. E: NR = 72 μm, ES = 101 μm.

**Figure 4:**
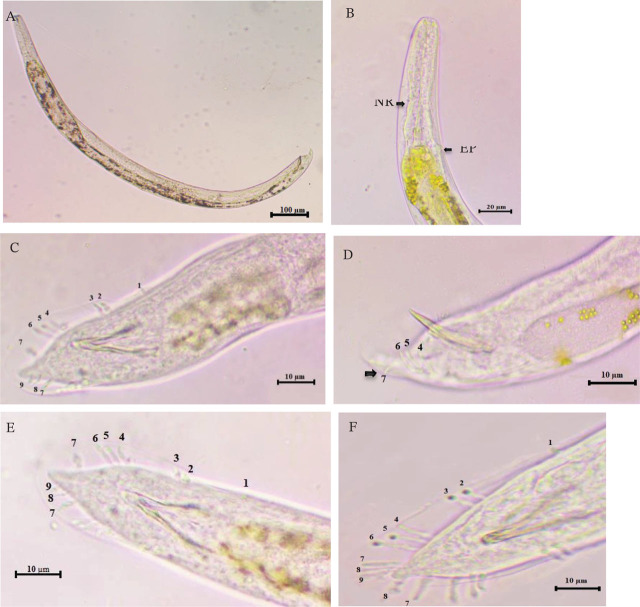

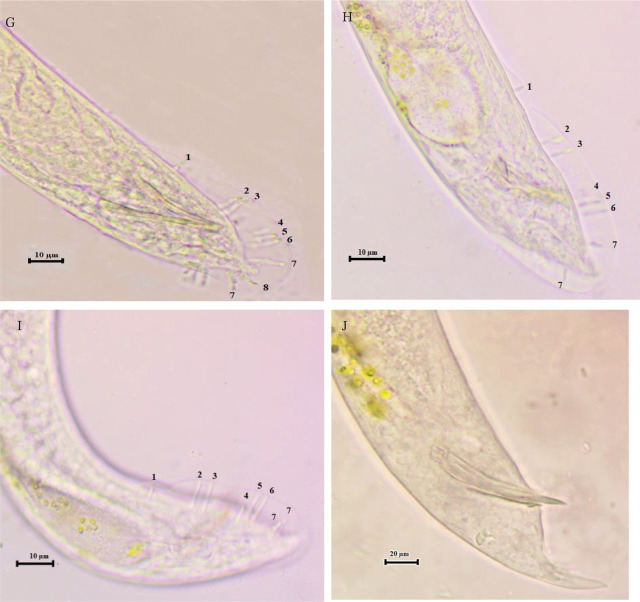
*Heterorhabditis pakistanense* male. A: Male entire body. B: The anterior portion; the left arrow refers to the nerve ring position, while the right arrow refers to the excretory pore. C, D: Male tail with bursal papilae. B, C: Ventral view of bursa showing papillae (3–2) in the terminal group with some variation in tall. D: Lateral view of bursa; the arrow refers to curved outward seventh pair. E, F, G, H: Lateral and ventral view of bursa showing (2–2) papillae in the terminal group, with some modifications (namely, being short and swollen). I: Lateral view of spicule and gubernaculm. J: Ventral view of spicule and gubernaculum. A = 705 μm. I, J: SL = 40 μm, GL = 19 μm.

Based on morphological characteristics and morphometric measures of male and infective juveniles, in addition to internal transcribed spacer (ITs) analysis, 14 out of the 27 isolates were identified as *H. indica* (Fig. 2), nine isolates as *H. bacteriophora* (Fig. 3), and two isolates as *H. pakistanense* (Fig. 4). Additionally, two isolates were identified as *S. affine,* the morphological, morphometric, and molecular characterizations of which have been documented in a separate scientific publication (Ali *et al*., 2022).

**Figure 5:**
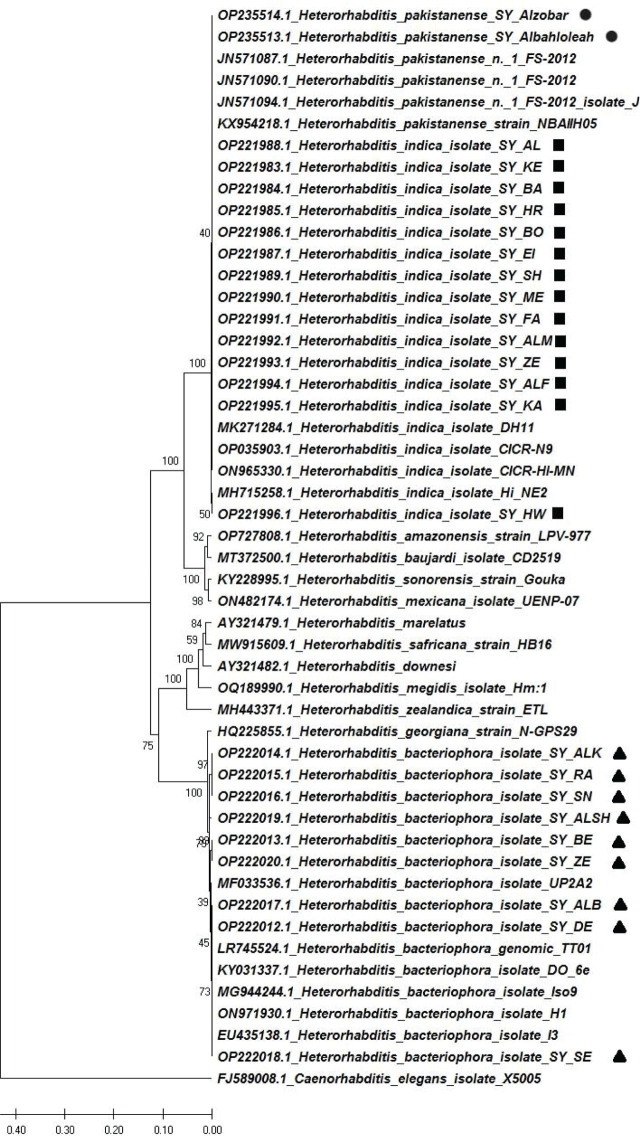
Evolutionary relationship of Syrian *Heterorhabditis* isolates using ITS rDNA sequences. The evolutionary history was inferred by using the maximum likelihood method. The bootstrap consensus tree inferred from 1,000 replicates is taken to present the evolutionary history of the analyzed taxa. *Caenorhabditis elegans* was used as an outgroup.

**Figure 6:**
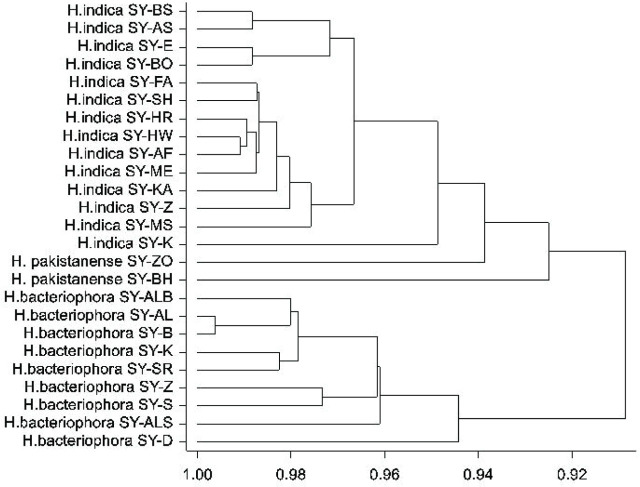
Cluster dendrogram of Syrian *Heterorhabditis* species isolates based on male morphological traits.

The morphological and morphometric characteristics of the Syrian heterorhabditid isolates were similar to the original descriptions provided by Poinar *et al.* (1992) for *H. indica* (Table 4 and Table 5), Poinar (1976) for *H. bacteriophora* (Table 6 and Table 7), and Shahina *et al.* (2016) for *H. pakistanense* (Table 8). According to the HCA analysis of selected males’ morphometric characteristics (Fig. 6), the Syrian heterorhabditid isolates displayed two main groups with bootstrap values ( > 94%). The first group included all *H. indica* isolates and the two isolates of *H. pakistanense.* All isolates of *H. bacteriophora* grouped together in a separate clade. On the other hand, the results of the phylogenetic tree analysis of the isolates, shown in Fig. 5, present the evolutionary descent among the different species, including the Syrian isolates in this study and those reported in the database. The analysis included four isolates from *H. indica*, seven isolates from *H. bacteriophora*, four isolates from *H. pakistanense*, and ten from different species.

**Table 4:** Morphometrics of infective juvenile of Syrian *Heterorhabditis indica* isolates. All measurements are in μm (except n, ratio, and percentage), and in the form: mean ± s.d. (range).

**Character**	**SY-BS**	**SY-E**	**SY-AS**	**SY-K**	**SY-BO**	**SY-FA**	**SY-SH**	**SY-HR**	**SY-KA**	**SY-Z**	**SY-MS**	**SY-ME**	**SY-HW**	**SY-AF**	***H. indica* Type strain Poinar et al. (1992)**
n	20	20	20	20	20	20	20	20	20	20	20	20	20	20	25
L	532±31 (503–589)	551±25 (532–591)	547±45 (523–598)	559±37 (518–589)	553±37 (510–595)	561±41 (529–602)	569±27 (520–596)	578±46 (545–610)	570±21 (531–604)	589±69 (520–610)	597±51 (532–614)	602±62 (541–610)	607±66 (539–615)	604±59 (551–618)	528 (479–573)
MBD	20.5±0.7 (18–22)	20±1.1 (19–23)	19±0.8 (17–21)	19.5±0.9 (18–22)	20 ±1 (18–21)	21±1.3 (17–24)	21±1.2 (18–23)	22±1.4 (18–24)	21.5±1.5 (19–24)	21±1.2 (18–25)	22±1.4 (17–24)	21±1.8 (18–26)	22±1.2 (19–25)	22±1 (18–25)	20 (19–22)
EP	97.2±6 (85–103)	95±5.5 (83–101)	94.5±5.3 (85–100)	96.5±5 (87–102)	94±4.9 (82–102)	96±5.2 (83–102)	101±6.1 (85–107)	98.5±4.7 (87–103)	101±4.5 (81–105)	102±6.7 (89–107)	102±6.4 (84–106)	101.5±58 (89–110)	103±6.5 (87–112)	102±6.3 (89–109)	98 (88–107)
NR	81.4±3 (70–86)	78.6±2.4 (74–87)	83±1.9 (78–88)	82.5±2.1 (73–86)	82±2.3 (75–84)	81±2.8 (70–85)	83±2.7 (74–88)	80±2.3 (75–86)	82.5±2.2 (73–85)	84±2.6 (74–88)	83.5±2.9 (71–87)	84±2.5 (75–88)	81±2.7 (72–86)	80.5±2.9 (75–84)	82 (72–85)
ES	118±4 (112–122)	117±5.1 (104–123)	118±6 (109–121)	117.5±5 (110–120)	115±5.6 (109–121)	121±5.4 (110–123)	121±5.8 (112–124)	122±7.4 (105–125)	118±6.1 (107–120)	119.5±6 (107–122)	120±6.1 (114–125)	119±5.4 (108–121)	118±5.7 (110–123)	120±5.5 (109–122)	117 (109–123)
ABW	9.6±1.1 (8–11)	7±0.7 (6.5–8)	7.1±0.8 (6.5–7.8)	7±0.7 (6.4–7.5)	6.7±1 (6.2–7.5)	7.2±0.8 (6.5–7.9)	7.5±0.9 (6.7–7.9)	7.2±0.4 (6.8–7.5)	7±0.6 (6.4–7.6)	7.8±0.9 (6.9–8)	6.8±0.8 (6.4–7.8)	6.7±0.9 (6.5–7)	7.1±0.5 (6.7–7.4)	7±0.5 (6.5–7.6)	-
T	103±5.1 (88–105)	94±2.3 (90–97)	96±4.1 (92–106)	97.5±2.3 (93–102)	97±1.4 (92–99)	98.5±2.9 (89–103)	95±2.1 (90–101)	102±5 (89–107)	98±4.9 (88–105)	101±4.1 (90–110)	98.5±4.5 (93–104)	102±4 (94–107)	104±4.3 (93–105)	101±4.9 (95–107)	101 (93–109)
a	25.9	27.5	28.7	28.6	27.6	26.7	27	26.2	26.5	28	27.1	27.3	27.5	27.4	26 (25–27)
b	4.50	4.7	4.63	4.75	4.82	4.63	4.70	4.81	4.83	4.90	4.97	5	5.14	5	4.5 (4.3–4.8)
c	5.1	5.86	5.69	5.73	5.70	5.69	5.98	5.66	5.81	5.83	6	5.90	5.83	5.98	5.3 (4.5–5.6)
D	0.82	0.81	0.80	0.82	0.82	0.79	0.83	0.80	0.85	0.85	0.85	0.85	0.87	0.85	0.84 (0.79–0.90)
E	0.94	1.01	0.98	0.98	0.96	0.97	1.06	0.96	1.03	1	1.03	0.99	0.99	1	0.90 (0.83–1.03)

**Table 5:** Morphometrics of male of the Syrian *Heterorhabditis indica* isolates. All measurements are in μm (except n, ratio, and percentage), and in the form mean ± S.D. (range).

**Character**	**SY-BS**	**SY-E**	**SY-AS**	**SY-K**	**SY-BO**	**SY-FA**	**SY-SH**	**SY-HR**	**SY-KA**	**SY-Z**	**SY-MS**	**SY-ME**	**SY-HW**	**SY-AF**	***H. indica* Type strain Poinar et al. (1992)**
n	20	20	20	20	20	20	20	20	20	20	20	20	20	20	12
L	685±89 (569–719)	697±80 (598–731)	689±75 (604–740)	715±69 (591–730)	703±81 (590–741)	718±87 (610–773)	726±89 (598–761)	741±91 (589–767)	735±85 (602–759)	749±80 (584–773)	738±90 (609–770)	745±77 (614–780)	754±94 (598–783)	762±79 (590–790)	721 (573–788)
MBD	37±2.3 (35–41)	38.5±2.4 (35–43)	37±2.1 (33–40)	41±1.5 (38–47)	40.5±1.8 (37–43)	41±1.3 (37–42)	41.5±1 (35–44)	42±1.2 (37–45)	42±1.9 (35–44)	43.5±1.6 (38–47)	43±0.9 (37–44)	44±1.4 (38–46)	43.5±1.5 (36–45)	43±1.9 (38–45)	42 (35–46)
EP	113±6.7 (105–125)	114.5±5.9 (107–121)	114±6.1 (107–127)	119±8.1 (107–127)	115.5±5.7 (109–125)	119±5.9 (105–130)	124±5.1 (109–131)	126±5.5 (110–137)	124.5±5.9 (105–134)	126±5.8 (114–136)	124.5±6.1 (107–133)	126.5±5.4 (112–136)	129±5.8 (110–139)	132±6 (109–140)	123 (109–138)
NR	71±4.5 (69–76)	72±5.8 (69–79)	71.5±4.3 (67–75)	74±4.7 (70–80)	72±6.1 (68–83)	73±5.7 (69–84)	73±4.1 (70–80)	78±5.5 (69–80)	77.5±4.3 (70–81)	78.5±4.9 (70–85)	77±3.9 (73–83)	78±4.5 (72–84)	79±5.1 (74–86)	78.5±5.2 (71–85)	75 (72–85)
ES	95±5.7 (94–104)	97.5±5.1 (92–103)	95.5±5.5 (93–105)	98±5.9 (92–107)	98.5±6.1 (94–107)	99±6.3 (95–108)	101±5.8 (95–107)	103±5.9 (94–106)	101.5±6 (96–109)	104±5.8 (94–107)	103±5.2 (96–105)	103.5±4.9 (95–106)	104.5±5.6 (94–109)	106±6 (97–109)	101 (93–109)
ABW	20±1.2 (18–22)	20±1.3 (17–21)	20.5±1.2 (18–23)	20±1.4 (17–23)	21±0.9 (18–24)	22±1.3 (19–24)	22.5±1.4 (18–24)	23±0.8 (19–24)	22±1.4 (19–25)	23±1.1 (17–25)	21.5±0.9 (19–24)	22.5±1.4 (18–25)	23.5±1.2 (18–25)	23±1.1 (18–25)	23 (19–24)
T	23±2.1 (21–26)	25±1.9 (22–29)	24.5±1.3 (23–30)	26±1.7 (21–30)	25±1.8 (22–30)	26.5±2.3 (24–33)	27.5±2.1 (24–31)	29±2.6 (21–32)	29±2 (24–31)	30±2.5 (22–32)	28±2.1 (24–31)	29.5±1.9 (24–32)	30±1.6 (24–31)	30±1.5 (25–33)	28 (24–32)
a	18.51	17.9	18.61	17.43	17.35	17.51	17.49	17.64	17.5	17.21	16.9	16.93	17.33	17.7	17.16
b	7.21	7.14	7.21	7.29	7.13	7.25	7.18	7.19	7.24	7.20	7.16	7.19	7.21	7.18	7.13
c	29.7	27.88	28.1	27.5	28.12	27	27.39	25.5	25.34	25.82	26.3	25.25	25.13	25.4	25.75
D	1.18	1.17	1.19	1.21	1.16	1.20	1.22	1.22	1.22	1.21	1.20	1.22	1.23	1.24	1.21
E	4.91	4.58	4.63	4.6	4.5	4.49	4.50	4.34	4.29	4.2	4.44	4.28	4.3	4.4	4.39
SL	37±3.9 (34–43)	38.5±3.7 (35–44)	37±3.5 (35–43)	41±4 (37–42)	39.5±3.2 (38–44)	42±2.1 (39–46)	43±3.1 (37–46)	44.5±3.4 (38–47)	43.5±3.2 (39–45)	45±3.9 (38–47)	44±3.1 (40–48)	44.5±3.2 (40–47)	46±3.8 (39–48)	47.5±4.1 (39–50)	43 (35–48)
GL	17.5±1.9 (17–22)	19±1.5 (17–22)	18±1.3 (17–21)	21±1.7 (19–22)	19.5±1.2 (18–22)	20.5±1.3 (19–23)	21±1.4 (17–22)	21.5±1.3 (19–22)	22±1.2 (19–23)	23±1.6 (19–25)	21±1.2 (20–24)	22±1.6 (19–23)	22.5±1.4 (19–24)	23±1.2 (18–24)	21 (18–22)
SW	1.85	1.92	1.80	2	1.88	1.90	1.91	1.93	1.89	1.95	2	1.97	1.95	2	1.86
GS	0.47	0.49	0.48	0.51	0.49	0.48	0.48	0.48	0.50	0.51	0.47	0.49	0.48	0.48	0.50 (0.40–0.60)

**Table 6:** Morphometrics of infective juvenile of the Syrian *Heterorhabditis bacteriophora* isolates. All measurements are in μm (except n, ratio, and percentage), and in the form mean ± S.D. (range).

**Character**	**SY-ALS**	**SY-SR**	**SY-ALB**	**SY-AL**	**SY-K**	**SY-S**	**SY-D**	**SY-Z**	**SY-B**	***H. bacteriophora* Type strain Poinar (1976)**
n	20	20	20	20	20	20	20	20	20	15
L	603±80 (532–613)	600±67 (521–611)	597±71 (527–608)	589±67 (529–603)	593±78 (522–610)	578±79 (524–602)	553±51 (519–597)	564±63 (520–602)	575±59 (518–590)	588 (512–617)
MBD	26±3.2 (18–33)	26±3 (17–31)	24.5±2.8 (18–29)	24±2.9 (18–31)	24±2.9 (18–33)	23.5±2.8 (18–31)	21.5±3.1 (18–29)	23±3.3 (18–31)	23±2.7 (18–31)	23 (18–31)
EP	106.5±5.4 (91–112)	106±5.6 (86–114)	105.5±6 (89–109)	102±6.3 (85–105)	104±6.8 (89–110)	98.5±5.7 (87–102)	93.5±5.1 (88–100)	94.5±5 (89–101)	97±5.5 (88–100)	103 (87–110)
NR	90±4.8 (75–95)	88.5±4.5 (72–92)	88.5±4.6 (72–90)	86.5±4.9 (74–90)	88±4.7 (71–94)	84±5 (72–90)	80.5±4.3 (70–91)	84±4.6 (71–92)	83±4.2 (70–89)	85 (72–93)
ES	132±6.2 (113–138)	131.5±5.7 (103–135)	129.5±5.9 (102–135)	126.5±6.1 (105–130)	128±6.3 (104–138)	124±6 (102–132)	114±5.9 (104–130)	119±5.6 (107–130)	122±6.2 (106–129)	125 (100–139)
ABW	8.2±1.2 (6–8)	8±1.1 (6.3–8.4)	7.8±0.9 (6.5–8)	7.4±0.7 (6.5–7.9)	7.5±1 (6.5–8)	7.2±0.7 (6.3–7.9)	6.5±0.9 (6.2–7.4)	6.8±0.9 (6.5–7.8)	7±1 (6–7.4)	-
T	108.5±6.7 (89–115)	107±6.4 (81–114)	104±6.1 (83–109)	100±5.8 (85–104)	102±6.2 (81–112)	95±6.3 (82–101)	89±6 (81–100)	92.5±5.6 (82–102)	93.5±5.1 (81–98)	98 (83–112)
a	23.1	23	24.36	24.5	24.7	24.59	25.72	24.52	25	25 (17–30)
b	4.56	4.56	4.61	4.65	4.63	4.66	4.85	4.73	4.71	4.5 (4–5.1)
c	5.55	5.60	5.74	5.89	5.81	6	6.21	6	6.14	6.2 (5.5–7)
D	0.80	0.80	0.81	0.80	0.83	0.79	0.82	0.79	0.79	0.84 (0.76–0.92)
E	0.98	0.99	1.01	1.02	1	1.03	1.05	1.02	1.03	1.12 (1.03–1.30)

**Table 7:** Morphometrics of male of the Syrian *Heterorhabditis bacteriophora* isolates. All measurements are in μm (except n, ratio, and percentage), and in the form: mean ± s.d. (range).

**Character**	**SY-ALS**	**SY-SR**	**SY-ALB**	**SY-AL**	**SY-K**	**SY-S**	**SY-D**	**SY-Z**	**SY-B**	***H. bacteriophora* Type strain Poinar (1976)**
n	20	20	20	20	20	20	20	20	20	15
L	845±91 (789–910)	829±85 (778–903)	810±65 (785–895)	819±69 (791–901)	824±84 (775–915)	831±95 (793–942)	804±79 (773–891)	820±83 (786–900)	815±74 (781–906)	820 (780–960)
MBD	46.5±4.8 (37–48)	44.5±5.1 (39–47)	41±4.1 (36–43)	41±4.5 (38–46)	42.5±4.6 (37–47)	44.5±4.1 (39–46)	40±4.2 (35–45)	42±4 (36–45)	41±4.3 (36–45)	43 (38–46)
EP	126±5.5 (115–129)	124±5.7 (114–126)	119.5±5.1 (115–125)	121.5±5.5 (115–125)	123±5.9 (115–128)	124.5±5.8 (115–130)	120±5.9 (112—124)	122.5±5.1 (116–128)	122±5.4 (114–129)	121 (114–130)
NR	76±4.7 (67–79)	73±4.6 (64–77)	69±4.1 (67–74)	71±4.5 (67–77)	72±5.1 (65–80)	73.5±5.2 (66–83)	68.5±4 (65–75)	71.5±4.3 (67–76)	71±4.7 (66–78)	72 (65–81)
ES	103.5±2.1 (100–104)	103±2 (99–104.5)	102±1.7 (99–104)	101±1.1 (100–103)	102.5±1.2 (98–104)	103±1.2 (99–104)	100±0.8 (98–102)	101±0.9 (99–103)	100.5±1 (97–104)	103 (99–105)
ABW	24.5±1.7 (22–26)	23±1.3 (21–24)	21±1.1 (20–23)	21.5±1.5 (20–24)	23±1.2 (21–24)	23.5±1 (21–25)	21±0.9 (20–23)	23±1.4 (20–24)	21±1.1 (20–24)	23 (22–25)
T	32.5±3.9 (24–34)	29±4.3 (22–32)	25±3.8 (24–30)	26.5±3.1 (24–31)	27±4.1 (22–34)	29.5±3.5 (24–35)	23.5±4.5 (21–30)	27±3.4 (24–30)	26±3.1 (24–31)	28
a	18.1	18.6	19.7	19.9	19.3	18.6	20	19.5	19.8	19
b	8.16	8.04	7.94	8.10	8.03	8.06	8.04	8.11	8.01	7.9
c	25.22	28.58	32.5	30.9	30.5	28.16	24.73	30.3	31.34	29.2
D	1.21	1.20	1.17	1.20	1.2	1.20	1.2	1.21	1.21	1.17
E	3.76	4.27	4.78	4.58	4.55	4.22	5.1	4.53	4.69	4.32
SL	42.5±2.5 (37–44)	40±2.6 (35–43)	37.5±2 (36–41)	39±1.7 (37–44)	39.5±1.8 (36–44)	41±2.9 (36–46)	37±2 (35–42)	39±1.7 (36–42)	39±1.6 (35–42)	40 (36–44)
GL	21±0.8 (19–23)	19±0.7 (18–23)	18±0.7 (17–22)	19±0.9 (18–23)	20±1 (17–23)	21±1.1 (18–24)	19±1 (17–22)	20±1.2 (17–23)	19±1.1 (17–23)	20 (18–25)
SW	1.73	1.73	1.78	1.81	1.71	1.74	1.76	1.69	1.85	1.74
GS	0.49	0.47	0.48	0.48	0.48	0.51	0.51	0.51	0.48	0.50

**Table 8:** Morphometrics of infective juvenile and male of the Syrian *Heterorhabditis pakistanense* isolates. All measurements are in μm (except n, ratio, and percentage), and in the form mean ± S.D. (range).

**Character**	***Heterorhabditis pakistanense* SY-BH**		***Heterorhabditis pakistanense* SY-ZO**		***Heterorhabditis pakistanense* n. sp. (Shahina *et al.*, 2016)**	
		
**Stage**	**Infective Juvenile**	**Male**	**Infective Juvenile**	**Male**	**Infective Juvenile**	**Male**
n	20	20	20	20	25	25
L	575±23 (536–597)	839.5±91 (710–945)	583±31 (542–603)	810±85 (721–991)	581 (558–624)	819 (720–1013)
MBD	20±2.7 (17–21)	39.5±2.4 (36–41)	19±0.8 (17–22)	38±2.1 (35–40)	21 (19–23)	39.8 (38–43)
EP	95.5±3.6 (85–98)	120.4±3.2 (113–129)	92±2.7 (87–98)	119±3.4 (114–125)	99.3 (95–106)	122.8 (112–133)
NR	83±2.5 (70–89)	87±3.8 (81–97)	81±1.5 (73–86)	85.6±4.2 (79–95)	82 (73–90)	89 (80–110)
ES	116.5±2.9 (109–121)	100.5±1.1 (97–103)	114±1.6 (107–119)	98.5±0.9 (97–101)	117 (113–125)	102 (100–105)
ABW	12.5±1.5 (9.4–15)	23.3±0.9 (21–25)	12±1.2 (10–15)	22.5±1.4 (21–26)	13.7 (12–16)	24.5 (22–26)
T	96.5±2.3 (92–103)	38±1.8 (32–41)	94±2.1 (90–105)	35.5±2.3 (31–40)	99 (95–110)	37 (30–42)
a	28.75	21.2	30.6	21.3	27.4 (25–29)	20.5 (18–23.5)
b	4.9	8.35	5.11	8.22	4.8 (4.7–5.3)	7.9 (7.2–9.8)
c	5.95	22	6.2	22.8	5.78 (5.4–6.2)	22 (19.3–25)
D	0.81	1.19	0.80	1.20	0.84 (0.78–0.97)	1.19 (1.1–1.26)
E	0.98	3.16	0.97	3.35	1 (0.95–1.07)	3.31 (2.67–4)
SL	-	39±2.4 (35–41)	-	37±2.7 (34–40)	-	38.5 (35–42)
GL	-	21.4±1.9 (19–23)	-	20.5±1.5 (19–22)	-	21 (20–22)
SW	-	1.67	-	1.64	-	1.56 (1.44–1.91)
GS	-	0.54	-	0.55	-	0.58 (0.48–0.65)

The results of the bursa study revealed variations and irregular arrangements of the terminal bursal ribs in *H. indica* (Fig. 2) and *H. pakistanense* (Fig. 4), while *H. bacteriophora*'s bursa appear normal and similar to the reference description (Fig. 3). In general, the numbers of genital papillae 1–6 remain unchanged and typical for *Heterorhabditis*: pair 1 is straight, reaching the bursal rim, and is located anterior to the spicule heads. Pairs 2 and 3 form a group located near the middle of the spicules that are straight and reach the bursal rim. Pairs 4, 5, and 6 pairs also form a group, located near the cloacal aperture. Laterally, the fourth pair turns outward and doesn’t reach the bursal rim, while the fifth and sixth pairs turn inward ventrally, and do reach the bursal rim.

In the 30 observed specimens, *H. indica* exhibited two forms of variations of the terminal group. The first form (63.33%) had three pairs (7, 8, and 9) on one side and two pairs (7 and 8) on the other side, while the second form (36.66%) had two pairs (7 and 8) on both sides. In both forms of the terminal bursal ribs, we observed that the papillae were sometimes modified to be short, narrow, and swollen at the base. We observed more variation in the terminal group of the *H. pakistanense* bursa. The bursa exhibited four distinct forms; the first form (50%), bursa had only one pair (7) on each side; in the second form (20%), bursa had three pairs (7, 8, and 9) on one side and one pair (7) on the other side; in the third form (16.66%), bursa had two pairs (7 and 8) on one side and one pair (7) on the other side; and in the fourth form (13.33%), bursa had three pairs (7, 8, and 9) on one side and two pairs (7 and 8) on the other side.

### Ecological trends of entomopathogenic nematode

Four species of EPNs were found in this investigation, as indicated in Table 1. *H. indica* was the most abundant species, representing 51.58% of the identified positive samples. It was isolated from citrus, olive, and walnut orchards, grassland, and vegetable fields during the autumn and spring seasons. *H. indica* was recovered from eleven sites in the Latakia governorate and from three sites in the Tartus governorate, across altitudes that ranged from 36 to 200 m, 200 to 400 m, and from 400 to 600 m. The soil textures included silty loam, sandy loam, sandy, and clay. Organic matter content (%) ranged from 1.4 to 5.72%, while pH levels varied from 5.2 to 8.1.

*H. bacteriophora* was detected in 33.33% of positive samples, primarily in sites located within Latakia governorate, at altitudes ranging from 4 to 200 m. A single occurrence was recorded at an elevation of 486 m in the Tartus governorate. *H. bacteriophora* was isolated from citrus orchards, grassland, tobacco fields, and vegetable fields during autumn and spring seasons, with soil texture including clay loam, silty loam, and sandy loam. Soil samples showed ranges from pH 4.8 to 7.8 and organic matter percentage between 1.25 and 6.2%.

*H. pakistanense* was isolated from 2 soil samples, representing 7.2% of positive samples. It was found to be associated with pear orchard and vineyard in the Latakia governorate during the autumn and summer seasons. The pear orchard and vineyard were at altitudes of 179 and 213 m, respectively, with a soil texture of sandy loam. The pH values were 6.1 and 7.1, while organic matter content was low, between 1.3 and 1.89%.

*S. affine* also was isolated from two soil samples, representing 7.2% of the positive samples. The samples were recovered from altitudes of 604 and 709 m in the Tartus governorate. *S. affine* was isolated from grassland and walnut orchards during the winter and spring seasons, respectively. The respective soil textures were silty and silty loam, with pH of 6.4 and 6.1, and organic matter content percentages of 4.1% and 6.1%.

**Figure 7:**
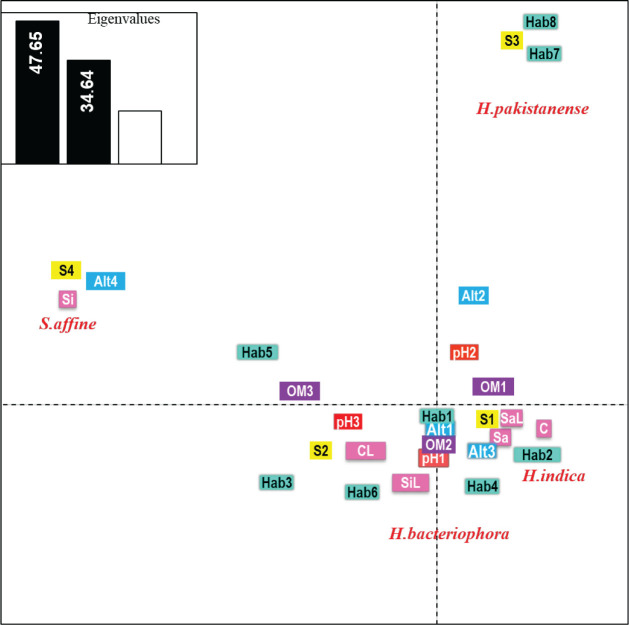
Co-inertia loading plot for EPN species and habitat and abiotic factors data in the Syrian coastal ecosystems surveyed.

The distribution of our EPN species was investigated according to habitat, soil characteristics, altitude, and sampling seasons. According to the co-inertia analysis (COIA), the fraction of variance accounted for by the two first COIA axes are 47.65 and 34.64% respectively (eigenvalues) (Fig. 7). The loading plot indicated an important contribution of *Heterorhabditis indica* (positive values, located on the right side of the plot) in opposition to *Steinernema affine* (negative values, located on the left of the plot), while, the axis COIA_2_ was essentially correlated with *H. pakistanense* (positive values), as opposed to *H. bacteriophora* (negative values). In correspondence with habitat and abiotic variables, the analysis also revealed distinct groupings among EPN species and the analyzed variables.

Both *H. indica* and *H. bacteriophora* showed close association with citrus orchards (Hab_1_), in altitudes from 0 to 200 m (Alt_1_), moderate organic matter levels of 2 to 4% (OM_2_), and acidic soil with a pH less than 6.5 (pH_1_).

More specifically, *H. indica* isolates appear to be associated with olive orchards (Hab_2_) and vegetables (Hab_4_), as well as clay, sandy and sandy loam soil types (C, Sa, and SaL), and the Autumn season (S_1_), *while H. bacteriophora* isolates were essentially correlated with tobacco fields (Hab_6_) and grassland (Hab_3_), clay loam and silty loam soils (CL, SiL), to alkaline soil higher than 7.5 (pH_3_), and to spring season (S_2_).

On the other hand, *Heterorhabditis pakistanense* isolates showed association with pear orchard and vineyard habitats (Hab_7_, Hab_8_), an altitude range of 200 to 400 m (Alt_2_), a moderate pH range of 6.5–7.5 (pH_2_), and a low organic matter content of 1 to 2% (OM1). *Steinernema affine* isolates were associated with walnut orchards (Hb_5_), silty soil type (Si), altitude of 600 to 800 m (Alt_4_) and winter season (S_4_).

### Virulence of native EPNs species against *T. absoluta*

**Figure 8:**
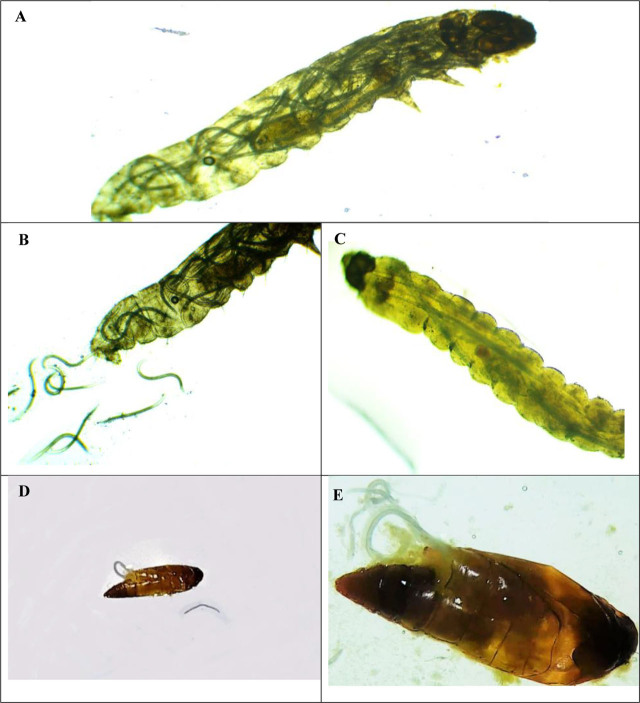
Larvae and pupae of *T.absoluta* infected by *S.affine* M.313. A: *S. affine* after completing its development within the fourth-instar larva. B: Emergence of the IJs from the infected fourth instar larva. C: Infected third instar larva. D, E: *S.affine* female inside pupae observed with the naked eye and 4× magnification.

**Figure 9:**
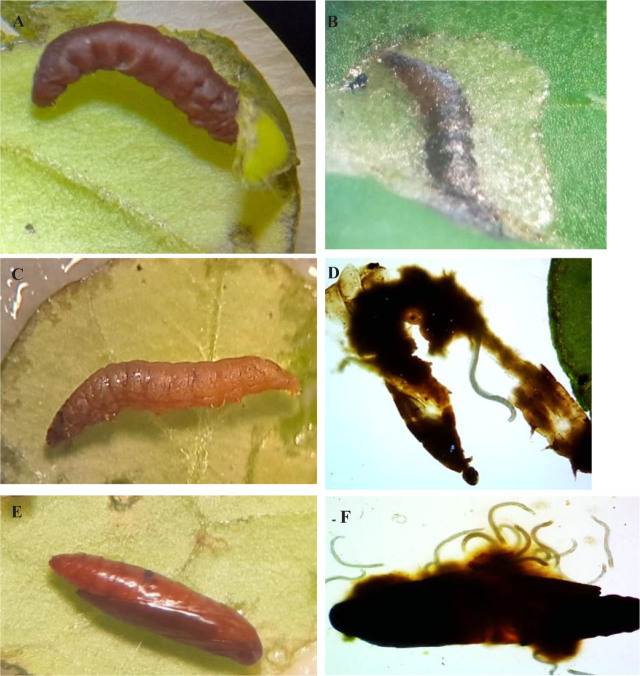
Larvae and pupae of *T.absoluta* infected by *H.indica* H. A, B: infected fouth-instar larvae outside leaf and inside mine. C, D: Infected third-instar larva outside leaf, and hermaphroditic inside it. E: Infected pupae. F: Hermaphroditics inside pupa.

The results obtained from experiments conducted on outside leaves showed that all three native EPN species tested had the capability to infect and kill the third- and fourth-instar larvae of *T. absoluta,* as presented in Table 9. However, the virulence varied among isolates, according to the applied dose of IJ/ larva. The mortality rates of the two tested instar larvae exhibited significant differences between the isolates on one hand and the control on the other, even at the low doses (1 IJ /larva). It is also noted from Table 9 that the mortality rate (%) gradually and significantly increases with the IJ dose increase. The isolate of *S. affine* M.313 showed the highest virulence for the third- and fourth-instar larvae (Fig. 8), followed by *H. indica* F. (Fig. 9), and then *H. bacteriophora* H. The mortality rates of the third-instar larvae of *S. affine* M.313 ranged from 25% at 1 IJ/larva to 79.17% at 50 IJ/larva, and from 29.17% to 91.67% for the fourth-instar larvae at 1 IJ/larva and 50 IJ/larva, respectively. *H. bacteriophora* H. exhibited the lowest mortality rates, even at the highest dose (50 IJ/ larva), with rates not exceeding 46% for the third-instar larvae, and 54% for the fourth-instar larvae.

**Table 9:** Mortality of third- and fourth-instar larvae of *Tuta absoluta* on outside leaves at each dose rate for three different entomopathogenic nematodes (mean ± S.D.).

	**EPNs species**	**Dose 1IJ/ L**	**Dose 5Ij/ L**	**Dose 10Ij/L**	**Dose 15Ij/ L**	**Dose 25Ij/ L**	**Dose 50Ij/ L**
Third-instar larvae of *T. absoluta*	*S. affine*. 313	25.00^a^±1.62	37.50^a^±2.16	45.83^a^±1.33	54.17^a^±1.45	66.67^a^±0	79.17^a^±0.95
	*H. indica*. Fn	16.67^b^±0	26.17^ab^±2.27	37.50^a^±1.33	45.83^a^±1.45	62.50^a^±1.43	70.83^a^±0.84
	*H. bacteriophora*. H	8.33^bc^±0.32	16.67^bc^±0.15	25.00^b^±2.62	33.33^b^±0	37.50^b^±1.43	45.83^b^±0.84
	Control	0^c^±0	0^c^±0	0^c^±0	0^c^±0	0^c^±0	0^c^±0
		*P*=0.00139^**^	*P*=0.0000657^***^	*P*=0.0000114^***^	*P*=0.0000105^***^	*P*=0.0000559^***^	*P*=0.0000011^***^
		df= 3	df= 3	df=3	df=3	df=3	df=3
		F=10	F=30.7	F=27.6	F= 65.33	F=108.5	F=97.22

Fourth-instar larvae of *T. absoluta*	*S. affine*. 313	29.17^a^±1.33	41.67^a^±1.64	50.0^a^ ±0	59.52^a^±1.16	71.43^a^±1.59	91.67^a^±1.62
	*H. indica*. Fn	20.83^ab^±1.33	36.50^ab^±1.33	41.67^ab^±1.62	50.0^ab^±0	50.00^a^±1.45	87.50^a^±1.33
	*H. bacteriophora*. H	12.50^ab^±2.17	25.00^b^±1.64	33.33^b^±0	41.67^b^±1.62	45.83^a^±1.33	54.17^b^±1.95
	Control	4.17^b^±1.62	4.17^c^±1.34	4.17^c^±1.33	4.17^c^±1.33	4.17^b^±1.33	4.17^c^±1.33
		*P*=0.00671^**^	*P*=0.000318^***^	*P*=0.0000175^***^	*P*=0.0000462^***^	*P*=0.00295^**^	*P*=0.0000311^***^
		df= 3	df= 3	df= 3	df= 3	df= 3	df= 3
		F=6.67	F=14	F=39.29	F=32.78	F=8.29	F=53.86

The mortalities including the same letter are not statistically different (*P* < 0.01).

According to the data analysis from the outside leaf assays, the LD_50_ for 3^rd^ instar larvae of *T. absoluta* was 15.85 IJ/larva for *S. affine* M.313, 24.19 IJ/larva for *H. indica* F, and 48.6 IJ/larva for *H. bacteriophora* H. For the fourth-instar larvae, LD_50_ was 11.38 IJ/larva for *S. affine* M.313, 15.71 IJ/larva for *H. indica* F., and 37.1 IJ/larva for *H. bacteriophora* H.

**Figure 10:**
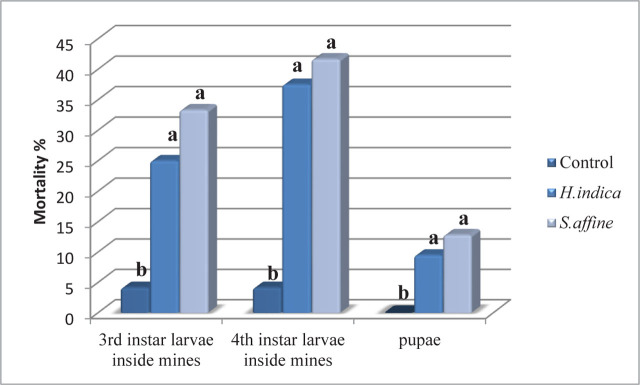
Mortality rates (mean ± SD) of third- and fourth-instar larvae inside the galleries and pupae of *T.absoluta* treated by LD_50_ inoculation rates of *S.affine* and *H.indica* (*P* = 0.01).

The results of inside mines assays of the third and fourth-instar larvae, as well as pupae, are presented in Fig. 10. Both *S. affine* M.313 and *H. indica* F showed the ability to effectively kill larvae within the mines; *S. affine* M313 caused mortality ranging from 33% for third-instar larvae to 42% for fourth-instar larvae. The mortality rate of *H. indica* F was slightly lower; it achieved 25% for third-instar larvae and 42% for fourth-instar larvae. No significant difference was observed between the EPN isolates studied, but the mortality rates caused were significantly greater compared to the control (*P* = 0.000932 and 0.000349, respectively). Considering the pupae assays, both EPN isolates demonstrated low mortality rates, at 12.83% for *S. affine* and 9.5% for *H. indica*. No significant difference was observed between the two isolates, or compared with the control (*P* = 0.05).

## Discussion

The current study represents the first systematic survey of the native EPNs in the Syrian coast regions. They were simply two local studies were conducted in the Latakia governorate, and were only intended to investigate the occurrence of EPN in a few stone-fruit orchards (Mosalam, 2009) and citrus orchards (Zeini *et al*., 2019). In this study, EPN were found in both governorates in the coastal regions. This contradicts the findings of Canhilal *et al*. (2006), but the reason probably the low number of samples collected in their study, which didn’t exceed 24 soil samples across all the coastal regions.

The prevalence of EPN in the soil samples in our research is low (3.28%). This result is not unusual and aligns with results from neighboring countries, such as 2% in Türkiye (Hazir *et al*., 2003), 0.9% in Jordan (Stock *et al*., 2008), 1% in Lebanon (Noujeim *et al*., 2011), and 3.2% in northwestern Iran (Eivazian Kary *et al*., 2009). Globally, the prevalence of EPNs varied widely, ranging from 0.7% to 100% (Bruck, 2004; Campos-Herrera *et al*., 2013). The recovery rates of EPN are influenced by a complex set of factors, some of them pertaining to environmental factors such as soil characteristics (Dzięgielewska and Skwiercz, 2018), while others relate to the methodology employed in sample collection and laboratory extraction. These could include sampling methods, the number and size of the samples, conducting random or targeted surveys, the type of insects used for baiting, laboratory temperature during isolation (Campos-Herrera *et al*., 2013; Tarasco *et al*., 2015b; Hazir *et al*., 2003).

On the other hand, EPN have a tendency to be aggregated in the soil in a more wilful than randomly distributed pattern because they are enormously reliant on insect aggregation (Campos-Herrera *et al*., 2012). EPN largely share this ecological niche with other organisms such as entomopathogens, which compete with them for limited resources (Hazir et al., 2022). In the present study, we discovered more than 200 isolates of entomopathogenic fungi (*Beauveria* spp., *Metarhizium* spp.). We recovered two isolates of *H. indica* in the same soil samples where the *Beauveria* spp were found. Interestingly, both isolates were obtained from only one *G. mellonella* larva between the 10^th^ and 12^th^ days after the beginning of the isolation process. We hypothesize that the decline in EPN recovery rates in our research may have also resulted from competition with entomopathogenic fungi (EPF), which were quite widespread within the soils of the studied sites on the Syrian coast. This competitive interaction leads to a reduction in host abundance. Consequently, when reaching a critical level of starvation, the IJ might best survive by becoming inactive until hosts become available again, as mentioned by Griffin (2015).

In this survey, the richness of the EPN species in the Syrian coast regions was low. Both *H. indica* and *H. bacteriophora* are widely distributed throughout the world (Bhat *et al*., 2020). *H. pakistanense* was discovered previously in Pakistan by Shahina *et al*. (2016), and in Syria for the first time during this survey, with a prevalence of 7.4%.

Additionally, *S. affine* was recorded for the first time in Syria in our previous study (Ali *et al*., 2022). This species is known to have spread to several European countries (Tarasco *et al*., 2015; Jaffuel *et al*. 2016) and has also been recovered in the Middle East, in Türkiye (Hazir *et al*., 2003). In this study, *H. indica* was detected for the first time in Syrian coastal regions and proved to be the most common species within the studied sites, as is consistent with previous studies (Abdel-Razek *et al*., 2018; Iraki *et al*., 2000)). In contrast, *H. bacteriophora* was prevalent in both local studies (Zeini *et al*., 2019; Mosalam, 2009), as well as many other investigations in Mediterranean and neighboring countries (Hazir *et al*., 2003; Stock *et al*., 2008; Eivazian Kary *et al*., 2009; Noujeim *et al*., 2011; Benseddik *et al*., 2020).

Although the topology and climate are similar for both targeted governorates, the occurrence of EPN in Latakia was higher than in the Tartus governorate. This is most likely due to the presence and spread of greenhouses in the coastal Tartus areas, of which there are estimated to be 141,735. In this agricultural ecosystem, the excessive use of conventional and novel chemical pesticides may have an impact on the viability of IJ, as mentioned by Laznik and Trdan (2013) and Ulu *et al*. (2016). Thus, all the soil samples collected from greenhouses were negative. In recent years, drought has become more frequent in Syria, so the difference also may be attributed to the spread of improved irrigation systems in the agriculture systems of Latakia, which, critically, provide moisture levels that more optimal for survival and persistence of nematodes in the soil. (Campos-Herrera *et al*., 2022).

In this study, the vegetation habitat had a significant effect on both the recovery frequency and abundance of nematodes. The frequency of positive soil samples was higher in undisturbed habitats compared to disturbed ones. We observed that citrus orchards and grassland, as well as olive orchards, supported high EPN abundance and activity. This result agrees with the hypothesis proposed by Campos-Herrera *et al*. (2013), which suggests a correlation between the presence and activity of EPN and rhizosphere stability in vegetation habitats like citrus orchards.

EPN also exhibited a higher recovery frequency at altitudes below 200 m. Within this altitude range, as revealed in the COIA analysis, *H. indica* and *H. bacteriophora* were dominant, which aligns with findings from other global studies (Hominick, 2002; Mauléon *et al*., 2006; Leonar *et al*., 2022) indicating that both species are commonly found at lower altitudes. Jaffuel *et al*. (2016) and Benseddik *et al*. (2018), however, reported that *H. bacteriophora* was recovered from high altitudes. *S. affine* was associated with high-altitude sites with low winter temperatures, significant snowfall, and moderate summer climates; this is consistent with Tarasco *et al*. (2015a), which recovered this species from high altitudes. *H. pakistanense*, was associated with altitudes ranging from 200 to 400 m. Notably, there was no previously available altitude data for this species, which was first discovered in Pakistan by Shahina *et al*. (2016).

In this survey, the pattern of EPN recovery was based on seasonal weather patterns. We found the recovery frequency of EPN was highest in the autumn, followed by the spring season. A similar result was reported by Campos-Herrera *et al*. (2010). The reason may be attributed to the nature of climate conditions in the Mediterranean region (moisture, rainfall, and temperature), which support the activity and survival of EPN populations, as well as the population of potential hosts in the spring and autumn seasons, the populations of which decline with the onset of the hot, dry season.

In general, a variety of factors can have an impact on the local distribution of EPN, such as soil texture, pH, organic matter content, and availability of hosts (Stuart *et al*., 2015; Hominick *et al*., 2002). Soil texture is one of the most important indicators affecting EPN distribution, and it accordingly plays an essential role in the longevity and persistence of dauer juveniles in natural ecosystems (Stuart *et al*., 2015). The majority of EPN-positive soil samples collected from Syrian coastal regions were identified as silty loam and sandy loam, and a few isolates were recovered from clay loam and clay soil. This is probably because soils with high sand/silt content favour EPN mobility and survival, while soils with high clay content restrict nematode movement (Shapiro-Ilan *et al*., 2012; Tarasco *et al*., 2015). Based on our results, it is worth noting that *H. indica* was less restricted by soil texture compared with other species, as it was isolated from a wide range of soil types, from sandy to clay. This aligns with previous research findings, which have also reported the presence of *H. indica* in various soil textures (Navarez *et al*., 2021; Mauléon *et al*., 2006).

pH levels can also significantly impact entomopathogenic nematode survival, so it is essential to consider this factor when selecting EPN species for biological control (Khathwayo *et al*., 2021). In our study, the pH levels of EPN-positive soils ranged from 5.2 to 8.1, falling within the survival range of pH 3 to 10 identified by Khathwayo *et al*. (2021). Organic material can improve the physical properties of the soil (e.g., bulk density, porosity, moisture–holding capacity), directly and indirectly affecting EPN (Stuart *et al*., 2015). In this study, the occurrence of EPN was observed in soils with varying organic matter content, ranging from low to high levels. This is consistent with many studies, including those by Miduturi *et al*. (1997) and Hazir *et al*. (2003).

For *Heterorhabditis*, morphological and morphometric traits are insufficient for species differentiation; they should be combined with the molecular characterization for the final confirmation of identity (Hominick, 2002). The present study reports the identification of three *Heterorhabditis* species from the Syrian coastal-region soils. By studying their morphological and molecular relationships, *H. pakistanense* (indica-group) was closely related to *H. indica*, as evidenced by the clustering dendrogram based on male morphometric traits. This similarity was also observed in the ML phylogenetic tree constructed using the ITS sequences. That was already reported by Shahina *et al*. (2016).

However, there are some differences between both species in the c value and E ratio of males; those of *H. pakistanense* were lower than those of *H. indica*. Conversely, the values of (a) and (b) were higher. On the other hand, it should be noted that compared to those in Shahina *et al*. (2016), the Syrian isolates of *H. pakistanense* showed more difference and variety in the number and arrangement of the males’ bursal terminal group papillae. The differences are thought to result from both intraspecific genetic variations and environmental factors.

The tomato leaf miner *Tuta absoluta* is one of the most destructive lepidopteran moths associated with tomatoes and other solanaceous plants in Syria., both in greenhouses and open fields. Controlling this pest has become more challenging due to its resistance to insecticides, which has resulted from its habit of feeding internally within plant tissues; high reproduction capacity; short generation cycle; and its reaction to intensive use of insecticides (Tarusikirwa *et al*., 2020; Gözel and Kasap, 2015). Based on the importance of directing efforts towards implementing biological control methods in conjunction with other strategies to establish a sustainable approach for managing *T. absoluta*, we conducted a virulence evaluation of three native EPN species in the genera *Heterorhabditis* and *Steinernema* against third- and fourth-instar larvae, as well as the pupae of *T. absoluta*. These isolates showed an ability to infect and kill larvae both outside and inside galleries with different levels of pathogenicity. However, their efficacy against pupae was limited. At 18 ºC, *S. affine* achieved the highest mortality rate across all assays, with *H. indica* closely behind. *H. bacteriophora* was the least efficient, possibly due to its poorer adaptation to cold in comparison to the other tested species. This result aligns with the research conducted by Van Damme *et al*. (2016), which indicated that *H. bacteriophora* demonstrated higher efficacy at 25 °C than at 18 °C.

The present study showed differences in susceptibility to EPN between larvae and pupae, with larvae being the most susceptible stage. These results are consistent with Türköz and Kaşkavalci (2016) and Samie *et al*. (2023). On the other hand, the mortality rates of fourth-instar larvae (both outside and inside galleries) were slightly higher than those in third-instar larvae. These results may be explained by a hypothesis, suggested by Van Damme *et al*. (2016), that the larger the host, the larger the natural openings in the host's body that commonly provide access to infective juveniles (IJ). Additionally, larger *T. absoluta* larvae emit stronger attractants, such as carbon dioxide (CO_2_), which in turn attract a greater number of IJ.

This comprehensive survey has provided valuable insights into the natural occurrence of entomopathogenic nematodes in Syrian coastal regions. Four EPN species, including three *Heterorhabditis* species and one *Steinernema* species, were identified. To the best of our knowledge, the isolation of *H. pakistanense* is the first such report from the Middle East. The recovery frequency of native EPN species was influenced by habitat, altitude, and sampling season. These native EPN species were detected in soils exhibiting diverse levels of organic matter content percentage and pH levels, with a notable prevalence in silty loam and sandy loam soils. *H. indica*, the most frequently identified species in this study, exhibited the most local adaptation to the environmental conditions of the region.

It will be necessary to conduct more in-depth research to gain a better understanding of the interaction between EPNs and other organisms within the soil, particularly with regard to entomopathogenic fungi (EPF) and its impact on EPN frequency in Syrian soils. The high efficacy of native EPN isolates observed in laboratory assays suggests potential as promising agents for safe and effective control of *T. absoluta*. However, future studies are recommended to assess their effectiveness against this insect in field conditions, in addition to evaluating their efficacy against other local pests in Syria.
